# Sequence Evolution and Expression of the Androgen Receptor and Other Pathway-Related Genes in a Unisexual Fish, the Amazon Molly, *Poecilia formosa*, and Its Bisexual Ancestors

**DOI:** 10.1371/journal.pone.0156209

**Published:** 2016-06-01

**Authors:** Fangjun Zhu, Ingo Schlupp, Ralph Tiedemann

**Affiliations:** 1 University of Evolutionary Biology/Systematic Zoology, Institute of Biochemistry and Biology, University of Potsdam, Potsdam, Germany; 2 Department of Biology, University of Oklahoma, Norman, Oklahoma, United States of America; Universitat de Barcelona, SPAIN

## Abstract

The all-female Amazon molly (*Poecilia formosa*) originated from a single hybridization of two bisexual ancestors, Atlantic molly (*Poecilia mexicana*) and sailfin molly (*Poecilia latipinna*). As a gynogenetic species, the Amazon molly needs to copulate with a heterospecific male, but the genetic information of the sperm-donor does not contribute to the next generation, as the sperm only acts as the trigger for the diploid eggs’ embryogenesis. Here, we study the sequence evolution and gene expression of the duplicated genes coding for *androgen receptors* (*ar*s) and other pathway-related genes, i.e., the *estrogen receptors* (*er*s) and *cytochrome P450*, *family19*, *subfamily A*, aromatase genes (*cyp19a*s), in the Amazon molly, in comparison to its bisexual ancestors. Mollies possess–as most other teleost fish—two copies of the *ar*, *er*, and *cyp19a* genes, i.e., *arα*/*arβ*, *erα*/*erβ1*, and *cyp19a1* (also referred as *cyp19a1a*)/*cyp19a2* (also referred to as *cyp19a1b*), respectively. Non-synonymous single nucleotide polymorphisms (SNPs) among the ancestral bisexual species were generally predicted not to alter protein function. Some derived substitutions in the *P*. *mexicana* and one in *P*. *formosa* are predicted to impact protein function. We also describe the gene expression pattern of the *ar*s and pathway-related genes in various tissues (i.e., brain, gill, and ovary) and provide SNP markers for allele specific expression research. As a general tendency, the levels of gene expression were lowest in gill and highest in ovarian tissues, while expression levels in the brain were intermediate in most cases. Expression levels in *P*. *formosa* were conserved where expression did not differ between the two bisexual ancestors. In those cases where gene expression levels significantly differed between the bisexual species, *P*. *formosa* expression was always comparable to the higher expression level among the two ancestors. Interestingly, *erβ1* was expressed neither in brain nor in gill in the analyzed three molly species, which implies a more important role of *erα* in the estradiol synthesis pathway in these tissues. Furthermore, our data suggest that interactions of steroid-signaling pathway genes differ across tissues, in particular the interactions of *ar*s and *cyp19a*s.

## Introduction

An estimated 25% of all plant species and 10% of animal species are involved in hybridization in nature [1,2]. Nonetheless, the role of hybridization in speciation has historically attracted little interest among zoologists, as hybridization has often been assumed to constitute a “reproductive mistake” [1,3], rather than a mechanism of speciation. In teleost fishes, hybridization among closely related species is relatively frequent, leading to increased genetic diversity and speciation [4,5]. Hybridization often compromises or even prevents further sexual reproduction, especially when two divergent parental homologs need to be paired during meiosis [6–9]. Such complications are circumvented, if hybrids shift to asexual reproduction. Most gynogenetic species originate from hybridization of bisexual ancestors [10,11]. Gynogenesis (also called sperm-dependent parthenogenesis or pseudogamy) is a peculiar mode of unisexual reproduction in which the sperm from a heterospecific donor is required for activating the development of the unfertilized diploid egg, yet the paternal genetic information does not contribute to the next generation [12]. Gynogenesis is enigmatic, as it seems to combine disadvantages of both asexual and sexual reproduction (i.e., no benefits from genetic recombination, but costs associated with mating).

The Amazon molly (*Poecilia formosa*) is a hybrid of the Atlantic molly (*Poecilia mexicana*, maternal) and the sailfin molly (*Poecilia latipinna*, paternal) originating from a single hybridization [13–20]. Mitochondrial DNA evidence suggests that the Amazon molly is at least 120,000 years old [21]. As an all-female and gynogenetic species, *P*. *formosa* occurs in a limited geographical range located along the coastal versant of northern Mexico, overlapping in distribution range with its sperm donor species [20]. Embryogenesis of diploid *P*. *formosa* eggs is triggered by sperm from closely related species (*P*. *latipinna*, *P*. *mexicana*, *P*.*latipunctata*) [13,22,23]. This reproductive mode involves copulation with heterospecific males, as egg development is initiated internally in *P*. *formosa*. In rare cases only, genomic material from the sperm enters the egg, leading to paternal introgression of either a complete haploid genome (causing triploids) or parts of a genome (creating microchomosomes) (for more details see ref [24,25]).

Sex hormones are crucially involved in a wide range of biological processes, including mating behavior, species recognition, regulation of reproductive behavior, and sexual development. Androgens, as a group of sex hormones, play a crucial role in stimulating the development of masculine traits, sex differentiation, mating behavior, and spermatogenesis in male vertebrates. Androgens are also indispensable for females [26,27]. A recent study reported that an increased production of 11-ketotestosterone was observed both in male and female *P*. *latipinna* after mating with conspecifics [28,29]. Interestingly, this was not detected in heterospecific matings between male *P*. *latipinna* and *P*. *formosa*, indicating a potential role of androgen bidirectional interactions in the recognition of conspecifics [28,29]. Males of both *P*. *mexicana* and *P*. *latipinna* have a preference to mate with their conspecific females, relative to mating with *P*. *formosa* [30,31]. However, the strength of this preference is weaker in *P*. *mexicana* than in *P*. *latipinna*.

Studies on other species have shown that the effect of androgens (i.e., testosterone (T) and 5α-dihydrotestosterone (DHT) in mammals, 11-ketotestosterone (11KT) and testosterone (T) in fish [27]) is mediated by androgen receptors (Ars), which belong to the nuclear receptor super-family [32]. In mollies, the genomic basis and the expression of androgen receptors has not been studied so far. Androgen precursors can be converted to estrogen, which plays a key role in reproduction and development by binding with the estrogen receptors (Ers). This conversion is catalyzed by cytochrome p450, family 19, subfamily A (Cyp19a) aromatase [33]. For Cyp19a and Ar, both of which are involved in high-affinity interactions in the steroid-signaling pathway, a coevolution has been reported in both vertebrates and invertebrates [34,35]. In teleost fish, coevolution of genes can be more complex, as almost all genes occur in duplication, due to the well-known whole genome duplication in fish.

In the present study, we investigate expression levels for six candidate genes among *P*. *formosa*, *P*. *mexicana*, and *P*. *latipinna* in different tissues, i.e., ovary, gill, and brain. Given the hybrid nature of *P*. *formosa* and its unisexual inheritance without meiosis, we aimed at evaluating gene-wise three alternative hypotheses, i.e., 1) genes in *P*. *formosa* maintain conserved absolute expression levels in different organs; 2) genes, which are involved in gonad differentiation and reproduction could be expressed differently in the unisexual hybrid, as compared to its bisexual ancestors. 3) gene expression levels in *P*. *formosa* reflect its hybrid nature, by either intermediate expression, relative to the ancestors or expression as one of the two ancestors, when ancestors differ in their expression pattern. We selected the target tissues for the following reasons: The brain, as an integration center, not only produces sex hormones needed for sex differentiation and reproduction, but also transmits the signals for appropriate behaviors to the whole organism [36,37]. The gills are considered to be a portal route between endogenous and exogenous sex hormones, as both males and females can release pheromonal compounds via the gills to communicate and stimulate each other [38–40]. Finally, the ovary is not only the organ for female gamete production, but also a major site of synthesis of sex hormones in females. It is also involved in hormonal regulation and endogenous interaction via the brain-ovary axis.

Our candidate genes comprise 3 pairs of gene duplicates, which have emerged in the course of the fish-specific whole-genome duplication (FSGD, [41]), i.e., two duplicates each of *ar*, *er*, and*cyp19a*. To our knowledge, for none of these genes, expression levels have been previously examined in these three species. We complement our study by sequence analysis of all genes. We investigate sequence evolution by comparison of inferred amino acid sequences with other vertebrate species.

## Materials and Methods

### Animals

Females from three species (*P*. *formosa*, *P*. *mexicana*, *P*. *latipinna*, at least four individuals per species) were used for this study. All specimens were laboratory born, fully sexual mature, and of similar body size. In order to avoid influences from interaction with male fishes, females were quarantined in separate tanks under standard conditions (12h light, 12h dark, 25°C) for 2 months. The *P*. *formosa* (For III/9) and *P*. *latipinna* (F.O II/7 1355) were kindly provided by Dr. Manfred Schartl, University of Würzburg. The founder fishes of the *P*. *formosa* were collected at Rio Purification (Barretal, Tamaulipas, Mexico) in 1993, *P*. *mexicana* (MIV/5) at Laguna de Champaxan (Altamira, Tamaulipas, Mexico) in 1994, and *P*. *latipinna* at Key Largo (Florida, USA) in 1993, respectively.

### Tissue Collection and RNA Extraction

All fishes were sacrificed on ice and all tissues were quickly excised, immediately moved into liquid nitrogen and then stored at -80°C. The whole procedure was accomplished in less than 15 minutes in order to minimize gene expression shifts. All procedures followed the international recognized guidelines and applicable national law (Tierschutzgesetz) and were approved by the deputy of animal welfare in Potsdam University. Our facility is approved for scientific work on fish by the regional veterinary official (amtliche Tierärztin, Stadt Potsdam). Sex and species assignment was re-confirmed by inspection of gonads and anal fin (for sex) and dorsal fin ray number (for species).

To increase total RNA yield, we performed a Trizol (LifeTechnologies) and RNeasy mini kit (Qiagen) combination RNA extraction method. The tissue was first homogenized in 1ml Trizol (as recommended by the manufacturer) by using a Mini-Beadbeater (Glen Mills Inc.). The aqueous phase from the centrifugation of the Trizol and chloroform mixture was transferred to an RNeasy mini kit column (Qiagen) and the downstream total RNA extraction and genomic DNA removal followed the RNeasy mini kit protocol including RNase-Free DNase (Qiagen). RNA concentration and quality was determined using a NanoDrop 1000 Spectrophotometer (ThermoScientific). RNA samples were stored at -80°C immediately after isolation.

### Reverse Transcription, Cloning and Sequencing

200ng RNA (DNase treated) of each sample was reverse transcribed using the RevertAid Fist Strand cDNA Synthesis Kit (ThermoScientific). In the negative control reaction for cDNA synthesis (-RT), the RT enzyme was replaced by water. For these control reactions, no amplification was observed neither in normal PCR nor in Real-Time PCR, such that no gDNA contamination was detected. We blasted full length sequences of our target genes of a phylogenetically related species, the Western mosquitofish (*Gambusia affinis*, Poeciliidae) [42,43] with *P*. *formosa* transcriptome data [44], and then designed primers for gene-specific amplification (see S1 Table for details). The target gene fragments were amplified with highly-reliable TopTaq DNA Polymerase (Qiagen). The *cyp19a2* gene was amplified using cDNA from brain, all other genes were amplified using cDNA from ovary tissue. Amplification parameters were: 30μl volume according to manufacturer specifications, 94°C for 120s, 38 cycles 94°C for 30s, 60°C for 30s, and 72°C for 2.5min. Amplificates were purified with the NucleoSpin Gel and PCR Clean-up kit (Macherey-Nagel). Cleaned fragments with the correct predicted length were then ligated into pCR™4-TOPO^®^ TA Vector which was used to transform One Shot TOP10 Electrocomp™ E. coli (Life Technologies). After positive kanamycin selection, fragments were re-amplified using T3/T7 PCR with Taq-polymerase (Taq Core Kit 10, MP Biomedicals Europe). PCR products were purified by using Exonuclease I and Antarctic Phosphatase (New England BioLabs) and sequenced on an ABI 3130xl automated sequencer (Applied Biosystems), using the BigDye Terminator v3.1 Cycle Sequencing Kit (Applied Biosystems). To facilitate the detection of both alleles in heterozygous state, we sequenced at least 12 clones for any gene of the gynogenetic species, *P*. *formosa*, and at least 6 clones for each bisexual ancestor species, *P*. *mexicana* and *P*. *latipinna*. PCR amplification from heterozygous specimens may be prone to template switching, leading to potentially chimeric sequences. In our analyses, this could potentially lead to chimera formation in the heterozygous *P*. *formosa*, i.e., an artificial combination of the alleles of *P*. *mexicana* and *P*. *latipinna* origin. We carefully checked our data for this phenomenon, but did not encounter such chimera in our cDNA-based sequence study.

The obtained sequences were aligned with sequences from other species (both teleost and other vertebrates) downloaded from the NCBI database (www.ncbi.nlm.nih.gov). The structural comparison domain analysis utilized both the Conserved Domain Database (CDD, NCBI) and Clustal Omega (EMBL-EBI, http://www.ebi.ac.uk/Tools/msa/clustalo). The alignment was compiled using Sequencher 5.2 (Gene Codes Corporation) and corrected manually. The final alignment was visualized in BioEdit [45]. The final amino acid alignment, initially constructed using MEGA V6.0 [46], was corrected manually and then used for construction of a phylogenetic tree with MrBayes (version 3.2.2, released August 16, 2013) (20 million generations, default settings [47]). The Jones-Taylor-Thornton (JTT) model [48] was the most suitable model determined by ModelGenerator (amino acid and nucleotide substitution model selection [49]). The final phylogenetic tree was produced in FigTree V1.4.2 (http://tree.bio.ed.ac.uk/software/figtree/). The functional implication of an inferred amino acid substitution was predicted using Polyphen2 (http://genetics.bwh.harvard.edu/pph2/) [50] and PROVEAN Protein (http://provean.jcvi.org/seq_submit.php) [51,52] online tools with the *P*. *latipinna* sequence as a reference. For those amino acid substitution in our study, for which Polyphen2 and or Provean indicated a departure from neutrality (i.e., predicted to be damaging/deleterious), we calculated variability, major AA frequency, and frequency of the specific AAs found in our alleles, by comparison to homologous sequences from GENBANK.

### Quantitative Real-Time PCR

Quantitative Real time PCR was carried out using an ABI 7500 Fast Real-Time PCR System (LifeTechnologies) in 20μl final volume, containing a 1:10 fold dilution of synthesized cDNA, 200nM of each primers (S1 Table) and SensiMix^TM^ SYBR Low-ROX kit (Bioline) with the following Parameters: Cycling Stage: 95°C for 10min, 40 cycles 95°C for 15s, 60°C for 1min; Melt Curve Stage: 95°C for 15s, 60°C for 1min, 1% increasing temperature up to 95°C for 30s, ending at 60°C for 15s. All candidate gene primers used for qRT-PCR were concentration-optimized and validated by sequencing. The qRT-PCR efficiencies (E) calculated by the 7500 software V2.0.1 (LifeTechnologies) were suitable for application of the Comparative CT (ΔΔ CT) method. The most stable reference gene, *rpl7* (Ribosomal Protein L7), was chosen for exploring the gene expression in various organs, after comparing it with Bestkeeper [53], NormFinder [54], and Genorm [55] to other extensively used reference genes in fish research, e.g. *gapdh*, *β-actin*, *tbp*, *hprt1* (data not shown). *rpl7* was also used as reference in previous expression studies on teleost fish [37,56]. The ΔΔ CT method [57] was used for expression analysis after the original CT values were normalized with the ExpressionSuit Software V1.0.3 (LifeTechnologies). CT values above 35 were omitted in our study and the Wilcoxon rank sum test was used for statistical comparison among expression patterns, as described in [37]. Three technical replicates from a single cDNA preparation from each specimen, were carried out in all real-time PCR experiments (see S2 File for experimental details; for the length and GC content of PCR products see S1 Table). All statistical analyses were performed in R, as well as figures were created using ggplot in the R package [58].

## Results

### Genetic variation and sequence evolution

**Fig 1:**
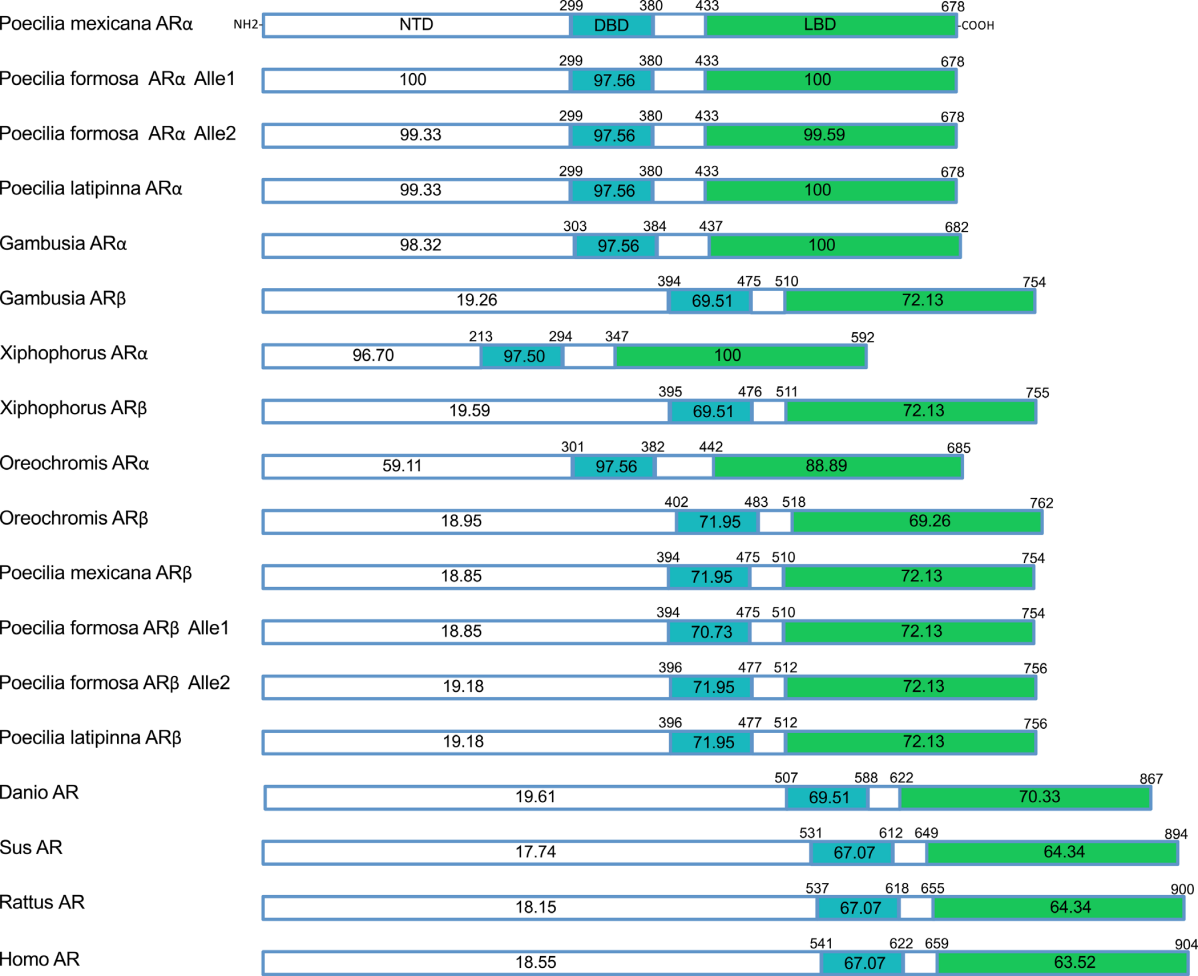
Structural comparison of *P*. *formosa* Ars with those of other species. The numbers within each box indicate the percent amino acid identity of each putative domain (NTD: N-terminal domain, DBD: DNA binding domain (blue color), LBD: ligand binding domain (green color)), relative to *P*. *mexicana* Arα. The numbers above each box indicate the amino acids position of each domain. *P*. *formosa* had two alleles of both Arα and Arβ, pointing to its hybrid origin.

As the consequence of a fish-specific, whole-genome duplication (FSGD [39]), *ar*, *er*, *cyp19a* have two isoforms in most teleost fish, i.e., *arα* and *arβ*, *erα* and *erβ1*, *cyp19a1* (also referred to as *cyp19a1a*) and *cyp19a2* (also referred to as *cyp19a1b*), respectively. In some teleost fish species, an additional isoform named *erβ2* has also been reported [59–62]. We initially cloned the entire coding region of both *ar* isoforms cDNAs, which contain different putative functional domains, namely the N-terminal domain (NTD), the DNA binding domain (DBD), and the ligand binding domain (LBD), as well as the entire coding region of *er*s and *cyp19a*s in our 3 focus species. All coding sequences are available from NCBI (for GenBank accession numbers see S1 Table). The Ar amino acids position of each putative domain is shown in Fig 1. The alignment of *Poecilia* Ars with those of other teleost fish and other vertebrate species illustrates that the important functional domains (e.g., DBD and LBD) are highly conserved, not only among the three species analyzed here, but also among Poeciliids in general (e.g., *Gambusia* and *Xiphophorus*; Fig 1, see S1 File for all pairwise comparisons).

**Fig 2:**
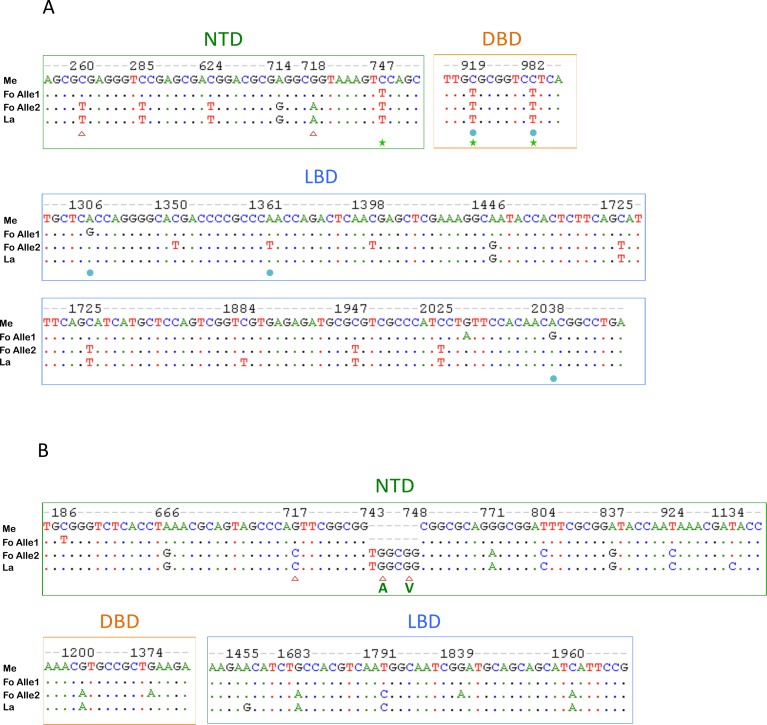
**Polymorphic nucleotide positions in the coding region of *arα* (A) and *arβ* (B) among the 3 analyzed *Poecilia* species.** Red triangles indicate non-synonymous substitutions among the ancestral alleles of *P*. *mexicana* and *P*. *latipinna* origin. The blue round point represents non-synonymous substitutions caused by mutation within a single lineage. The green capital letters below the alignment in NTD of *arβ* represent additional amino acids at a position with an insertion/deletion event. Green stars indicate positions, at which *P*. *mexicana* was heterozygous (C/T). Dots in the alignment indicate identity with *P*. *mexicana*. Colored boxes indicate different functional domains within the protein (Green: NTD, Blue: LBD, Orange: DBD). Abbreviation in figure, Me: *P*. *mexicana*, Fo: *P*. *formosa*, La: *P*. *latipinna*.

Due to its hybrid origin and subsequent unisexual inheritance, *P*. *formosa* has been previously reported to be fixed in a heterozygous state (frozen hybrid) at many loci [19,63]. Indeed, the alignment of the CDS of *P*. *formosa ar*s with its ancestor species also shows a heterozygous state of *ar* in *P*. *formosa* (Fig 2). Based on our *arα* and *arβ* sequences, *P*. *formosa* inherited one allele per locus from its maternal ancestor (*P*. *mexicana*), the other from its paternal ancestor (*P*. *latipinna*). In the coding region of *arα*, there are nine polymorphisms distinguishing between the two parental lineages, including five polymorphisms in NTD, four in LBD, but none in DBD. In the alignment of *arβ*, the two parental lineages are distinguished by ten differences distributed in all three domains and a 6bp insertion/deletion. Non-synonymous differences among the parental lineages were only observed in the NTD of *ar*s, but not in the functional domains. This implies that there is no diversity in the functional domain amino acid sequences, which suggests that proteins of all species maintain a similar biological function (e.g., downstream binding). We conducted the same analysis on *er*s and *cyp19a*s CDS (S1 Fig). Interestingly, we observed only one allele of *erβ1* in *P*. *formosa* transcripts, which originated from *P*. *latipinna*. A prediction of potential functional implications of non-synonymous substitutions is shown in Table 1, using two different prediction methods, i.e., Polyphen2 and PROVEAN. Consistently inferred by both methods, there are two sites (positions 919 and 982, corresponding to amino acid positions 307 and 328) inferred to exhibit a potential functional change in Arα. At both these sites, *P*. *formosa* shared a nucleotide (and inferred AA) with *P*. *latipinna* (putatively ancestral), while the character state in *P*. *mexicana* was different (putatively derived). The non-synonymous substitutions specific to *P*. *formosa* in Ars are generally inferred not to cause a functional change, except for the AA position at 454 predicted to have functional implications by the PROVEAN method. The non-synonymous single nucleotide polymorphisms (SNPs) among the ancestral bisexual species (interpreted as ancestral substitutions; cf. Table 1) in all genes were predicted not to alter protein function. This suggests that the heterozygous state of *P*. *formosa* at these sites is not harmful to the biological function of the respective proteins. For AA substitutions inferred to deviate from neutral expectations (i.e., inferred to be probably damaging/deleterious), variability, major AA frequency, and frequency of the specific AAs found in our alleles is provided in S2 Table. All but two of these substitutions occured at highly conserved positions (major allele frequencies of 74%-100%; S2 Table).

**Table 1:** Prediction of impact of non-synonymous inferred amino acid substitutions (cf. Fig 2).

Impact of amino acid substitution										
					Poly	Poly	Poly	Poly	PROV	PROV
	Site	Code	Pm-Pf1-Pf2-Pl	Interpretation#	Score	Sensitivity	Specificity	Prediction	Score	Prediction (cutoff = 0.25)
	260	V87A	A-A-V-V	ancestral	0.104	0.93	0.86	BENIGN	0.471	Neutral
	718	S240G	G-G-S-S	ancestral	0.000	1.00	0.00	BENIGN	0.971	Neutral
	919	C307R	R-C-C-C	derived in Pm	0.993	0.70	0.97	PROBABLY DAMAGING	-10.455	Deleterious
**Arα**	982	F328L	L-F-F-F	derived in Pm	0.944	0.80	0.95	PROBABLY DAMAGING	-5.374	Deleterious
	1306	T436A	T-A-T-T	derived in Pf	0.028	0.95	0.81	BENIGN	-1.255	Neutral
	1361	Q454L	Q-Q-L-Q	derived in Pf	0.002	0.99	0.30	BENIGN	-6.024	Deleterious
	2038	T680A	T-A-T-T	derived in Pf	0.001	0.99	0.15	BENIGN	-0.259	Neutral
	717	Q239H	Q-Q-H-H	ancestral	0.024	0.95	0.81	BENIGN	-0.753	Neutral
**Arβ**	743–748	247,AV,.	..-..-AV-AV	ancestral	-	-	-	-	0.968	Neutral
	286	T96A	A-A-T-T	ancestral	0.000	1.00	0.00	BENIGN	0.097	Neutral
	517–534	G173_V178del		ancestral	-	-	-	-	4.068	Neutral
	545	V182A	A-A-V-V	ancestral	0.000	1.00	0.00	BENIGN	0.681	Neutral
	551	V184A	A-A-V-V	ancestral	0.000	1.00	0.00	BENIGN	0.782	Neutral
	554	G185A	A-A-G-G	ancestral	0.003	0.98	0.44	BENIGN	0.341	Neutral
**Erα**	847	T283A	A-A-T-T	ancestral	0.001	0.99	0.15	BENIGN	-0.130	Neutral
	961	S321G	G-G-S-S	ancestral	0.000	1.00	0.00	BENIGN	-1.389	Neutral
	997	G333S	S-S-G-G	ancestral	0.402	0.90	0.90	BENIGN	0.472	Neutral
	1364	E455G	E-E-G-E	derived in Pf	1.000	0.00	1.00	PROBABLY DAMAGING	-5.836	Deleterious
	1523	S508L	L-L-S-S	ancestral	0.014	0.96	0.79	BENIGN	-3.812	Deleterious
	1602	M534I	I-I-M-M	ancestral	0.416	0.89	0.90	BENIGN	-2.848	Deleterious
	1679	R560H	H-H-R-R	ancestral	0.001	0.99	0.15	BENIGN	-1.931	Neutral
	1724	K575I	I-I-K-K	ancestral	0.001	0.99	0.15	BENIGN	-0.593	Neutral
	1815	P606A	P-A-P-P	derived in Pf	0.710	0.86	0.92	POSSIBLY DAMAGING	-0.501	Neutral
	272	N91S	S-S-N	derived in Pl	0.001	0.99	0.15	BENIGN	0.167	Neutral
	328	G110S	S-G-G	derived in Pm	0.007	0.96	0.75	BENIGN	0.510	Neutral
	409	A137T	T-A-A	derived in Pm	0.017	0.95	0.80	BENIGN	-0.096	Neutral
**Erβ1**	1322	A441G	G-A-A	derived in Pm	0.999	0.14	0.99	PROBABLY DAMAGING	-3.046	Deleterious
	1510	I504V	V-I-I	derived in Pm	0.893	0.82	0.94	PROBABLY DAMAGING	0.285	Neutral
	1584	A528del	.-A-A	derived in Pm	-	-	-	-	-0.186	Neutral
	1622	A541G	G-A-A	derived in Pm	0.000	1.00	0.00	BENIGN	-0.717	Neutral
	39	T13N	N-N-T-T	ancestral	0.007	0.96	0.75	BENIGN	-0.209	Neutral
**Cyp**	52	G18S	S-S-G-G	ancestral	0.078	0.93	0.85	BENIGN	0.625	Neutral
**19a1**	76	L26I	I-I-L-L	ancestral	0.000	1.00	0.00	BENIGN	0.021	Neutral
	899	G300D	G-G-D-G	derived in Pf	0.836	0.84	0.93	PROBABLY DAMAGING	-5.219	Deleterious
	1048	V350I	I-I-V-V	ancestral	0.001	0.99	0.15	BENIGN	-0.597	Neutral
	31	D10E	E-E-D-D	ancestral	0.002	0.99	0.30	BENIGN	0.222	Neutral
	64	V22L	V-V-L-L	ancestral	0.000	1.00	0.00	BENIGN	0.146	Neutral
	72	L27del	L-L-L-.	derived in Pl	-	-	-	-	1.088	Neutral
	101	L33P	P-L-L-L	derived in Pm	0.974	0.76	0.96	PROBABLY DAMAGING	-2.353	Neutral
	121	A40T	T-T-T-A	derived in Pl	0.000	1.00	0.00	BENIGN	0.623	Neutral
**Cyp**	246	A82T	T-T-A-A	ancestral	0.000	1.00	0.00	BENIGN	0.029	Neutral
**19a2**	410	T136I	I-I-T-T	ancestral	0.000	1.00	0.00	BENIGN	1.34	Neutral
	766	S255R	R-R-S-S	ancestral	0.003	0.98	0.44	BENIGN	-0.725	Neutral
	791	I263N	N-N-I-I	ancestral	0.023	0.95	0.81	BENIGN	0.906	Neutral
	802	T267A	A-A-T-T	ancestral	0.000	1.00	0.00	BENIGN	1.485	Neutral
	879	E292D	D-D-E-E	ancestral	0.000	1.00	0.00	BENIGN	-0.595	Neutral
	1019	D339V	V-V-D-D	ancestral	0.010	0.96	0.77	BENIGN	-1.346	Neutral

Two prediction methods, Polyphen2 (Poly) and PROVEAN Protein (PROV), were applied to assess the impact of inferred amino acid substitutions in the duplicated androgen receptor genes among the 3 species. The code provides details on the amino acids substitution/insertion and the its position. Pm-Pf1-Pf2-Pl provides the respective amino acid of *P*. *mexicana (Pm)*, *P*.*formosa* allele1 (Pf1), *P*. *formosa* allele2 (Pf2) and *P*. *latipinna* (Pl). Site: Polymorphic nucleotide position. indicates lacking amino acid positions due to an insertion/deletion event

# see discussion for details

**Fig 3:**
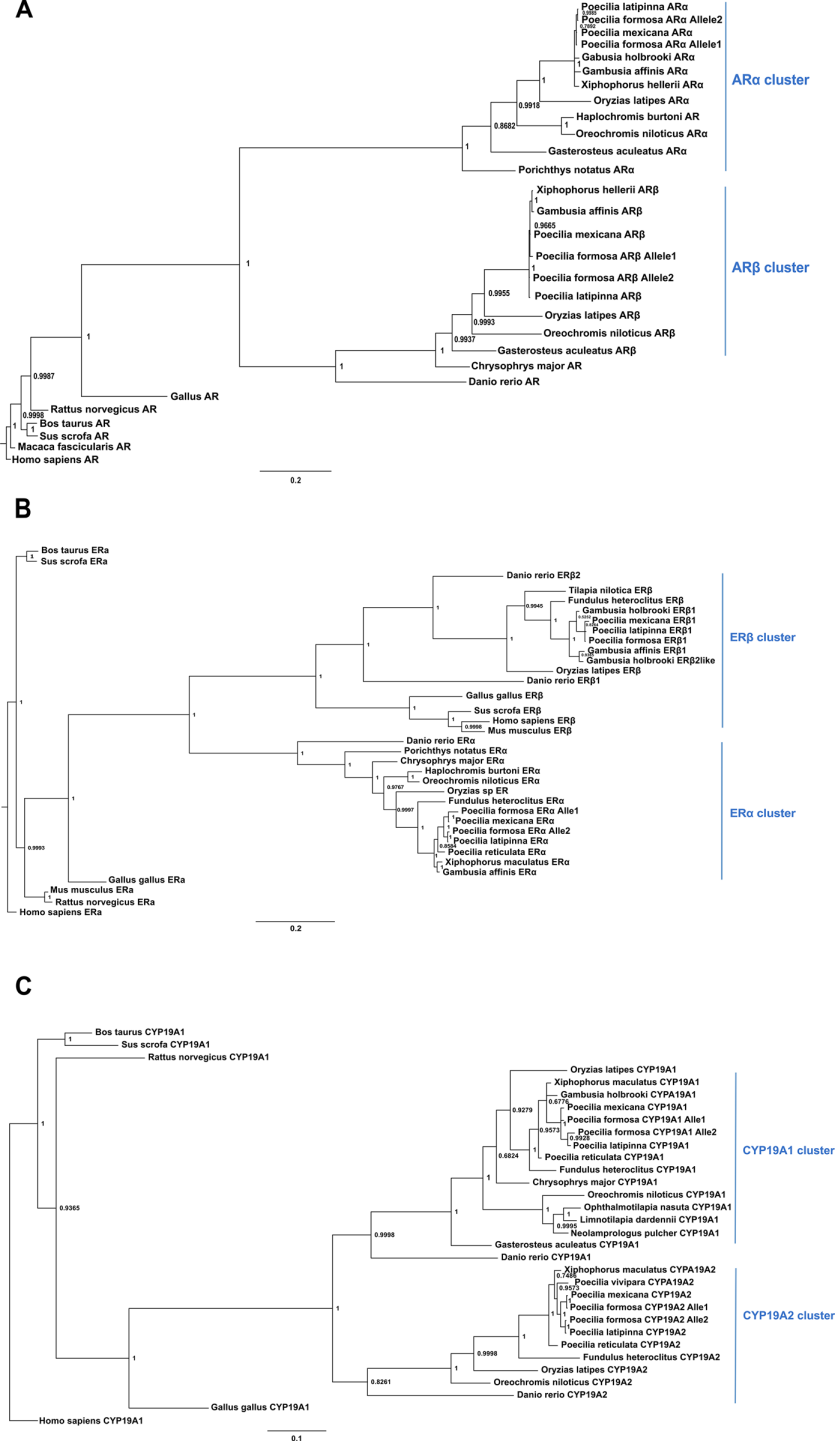
**Phylogenetic tree of *ar*s (A), *er*s (B) and *cyp19a*s(C) in Poeciliidae and other vertebrates.** Amino acid sequences were used for phylogenetic analysis with MrBayes. Labels provide Bayesian posterior probabilities. Teleost fish typically possess two copies of all genes. Note that *Danio rerio* has lost *arα*. This phylogenetic tree clearly demonstrates that the two gene copies emerged in the course of the fish-specific whole-genome duplication (FSGD, Meyer and Schartl 1999). It also confirms the hybrid origin of *P*. *formosa*, as they are heterozygous and possess one allele per locus from each of its ancestors. See S1 Table for accession numbers.

To obtain a more detailed understanding of *ar*s and related pathway genes sequence evolution in Poeciliidae, we constructed a phylogenetic tree based on the CDS of candidate genes using MrBayes (Fig 3). The phylogenetic tree clearly illustrates that the *ars* of fish fall into two clusters, *arα* and *arβ*. Furthermore, the *arβ* of fish is–on average—quite similar to the *ar* of other vertebrates or mammals. Our phylogenetic tree clearly uncovers a heterozygous state for *P*. *formosa* with allele1 inherited from *P*. *mexicana* and allele2 from *P*. *latipinna* on both the *arα* and *arβ* gene, confirming its hybrid origin once again. The high similarity of *ar*s across Poeciliidae (i.e., genera *Poecilia*, *Gambusia*, and *Xiphophorus*) is also illustrated in our phylogenetic tree. The other duplicated candidate genes exhibited a similar pattern and clustered consistently with a previous study [37]. Note that for *erβ1*, only one allele was revealed in *P*. *formosa* transcripts, i.e., that originating from *P*. *latipinna* (S1 Fig). For ARβ, the gene tree does not fully resemble the species tree of the respective *Poecilia* species. Inspection of the data revealed that this pattern is supported by the AA polymorphism at position 239 where *P*. *latipinna* and *P*. *formosa* allele2 show Histidine, but other related species (including *P*. *mexicana*) Glutamine.

### Expression profiles of candidate genes

**Fig 4:**
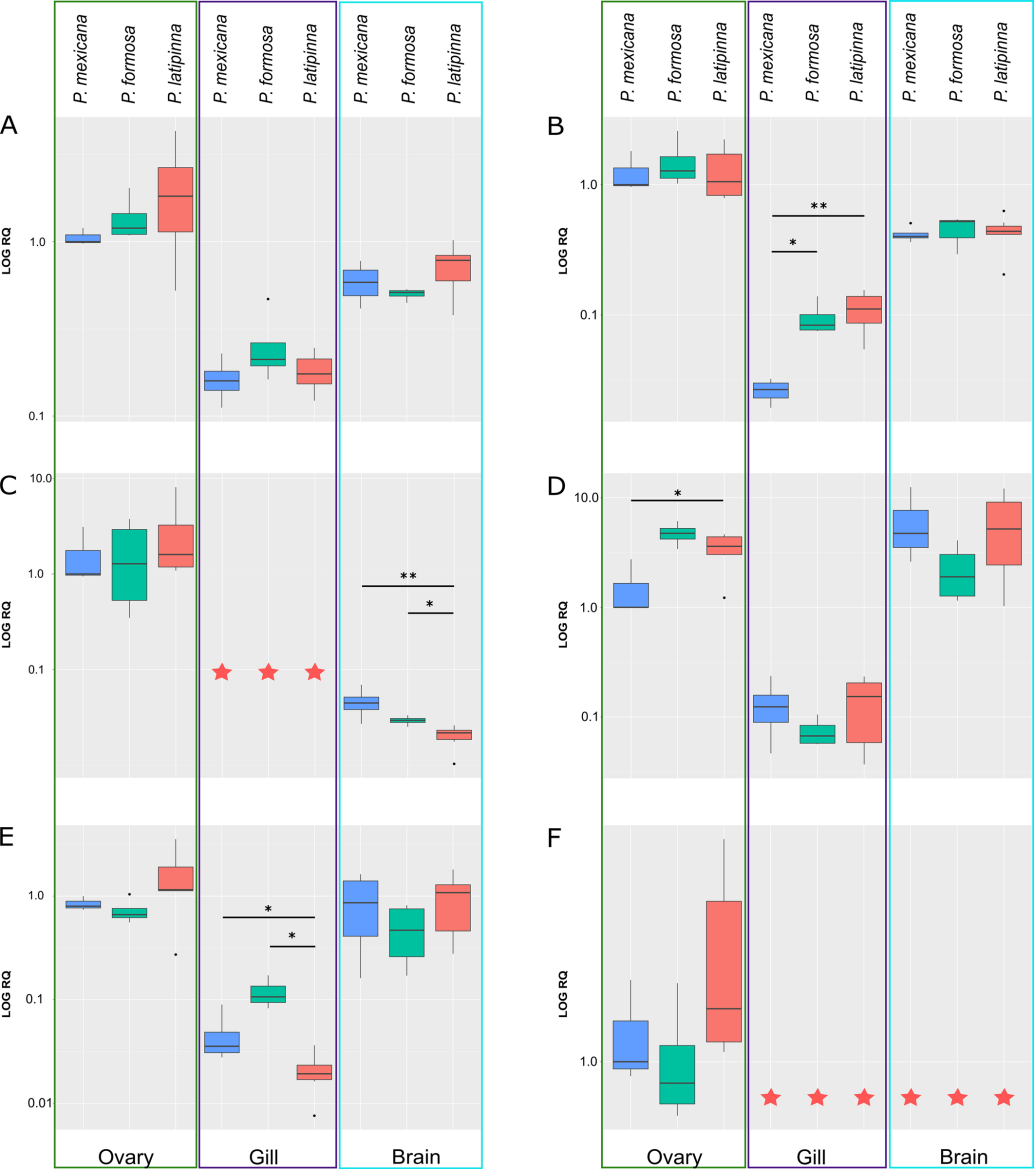
Gene expression profiling. This profiling presents each single gene expression distribution through all 3 tissues (ovary, gill, brain) among *P*. *formosa* and its ancestors. Expression levels are normalized relative to *P*. *mexicana* ovary expression data of 6 different pathway genes, *arα* (A), *arβ* (B), *cyp19a1* (C), *cyp19a2* (D), *erα* (E), *erβ1* (F). They are shown on a logarithmic scale. Red stars indicate that expression has not been detected in our study. * 0.01≤P≤0.05; ** P< 0.01.

In order to identify potential differences in gene expression between the gynogenetic hybrid species *P*. *formosa* and its bisexual ancestors, *P*. *mexicana* and *P*. *latipinna*, we performed quantitative reverse transcriptase-polymerase chain reaction (qRT-PCR) on six candidate genes which are involved in the sex hormone-related pathways on RNA isolated from ovary, gill, and brain tissue in 3 species. Differences in expression patterns across tissues are shown in Fig 4 (for relative expression value and statistical analysis results see S2 File). Generally, expression levels were the lowest in gill. The highest values were obtained in the ovary, except for *cyp19a2* (in *P*. *mexicana* and *P*. *latipinna*) and *erα* (in *P*. *mexicana*) where the highest expression was measured in brain. Fig 4 also provides detailed information on expression variation among genes, tissues, and species, as well as among biological replicates.

The general expression pattern of *ar* genes (Fig 4A and 4B) implies that the gynogenetic *P*. *formosa* maintains a conserved expression similar to its bisexual ancestors for the crucial genes involved in the sex hormone function pathways. No specifically reduced or absent expression in any gene or tissue was observed in *P*. *formosa*, the all-female species.

In two cases, *arβ* in gill and *cyp19a2* in ovary, *P*. *mexicana* had a significantly lower expression than the other two species. Likewise, in two other cases, *cyp19a1* in brain and *erα* in gill, *P*. *latipinna* had a significantly lower expression than the other two species. In summary, (1) there were only four genes with a significant difference among the two bisexual ancestral species in one of the analyzed tissues and (2) for these genes and tissues, the hybrid species *P*. *formosa* always exhibited an expression level alike the higher expression level among the two bisexual species. In three cases, *cyp19a1* in gill and *erβ1* in gill and brain, no expression was detected in any of the analyzed species.

## Discussion

In this study, we investigated sequence evolution in the genes of the androgen receptor and related pathways. Both bisexual molly species were found to be fixed for a single allele in our laboratory strains in all candidate genes (except for the *arα* gene in *P*. *mexicana* where 3 Single Nucleotide Polymorphisms (SNPs) occured; cf. Fig 2), while the hybrid Amazon molly was heterozygous with one allele per gene clearly deriving from *P*. *mexicana*, and the other from *P*. *latipinna* (except for the *erβ1* gene). Consequently, we tentatively suggest that a polymorphism predates the formation of *P*. *formosa* (called “ancestral” in Table 1), if we distinguish between (1) *P*. *latipinna* and the *P*. *latipinna*-derived allele of *P*. *formosa* and (2) *P*. *mexicana* and the *P*. *mexicana*-derived allele of *P*. *formosa*. If a substitution occurs only in one of the three species, it may have occurred after the formation of *P*. *formosa* (called “derived” in Table 1). For the *erβ1* gene, only one allele was found in *P*. *formosa* transcripts, despite of the analysis of 26 clones from that cDNA amplificate in our study. The likelihood for having overlooked a second allele is very low (P = 3.414E-07, Χ^2^ test). This might indicate that only one allele is present in *P*. *formosa* (e.g., because of gene conversion, cf. [19]). Alternatively, as our study is based on cDNA, it may have been caused by different allelic specific expression (ASE). Although the single allele in *P*. *formosa* likely derived from *P*. *latipinna*, its expression pattern did not show a larger deviation from the expression in *P*. *mexicana*. Expression of *erβ1* was very low in the ovary and not detected in gill and brain in all species (more intensely discussed below). Among the inferred amino acid substitutions, those with a higher predicted impact on protein function (i.e., predicted as “probably damaging” by Polyphen2 and/or “deleterious” by PROVEAN; cf. Table 1) were all found deviant (“derived” in Table 1) in the ancestral species *P*. *mexicana*, while those substitutions distinguishing between the two bisexual species (“ancestral” in Table 1) and specific to *P*. *formosa* (except AA position 454 by PROVEAN method) were mostly predicted to be benign or neutral. This suggests that the heterozygous state of *P*. *formosa* is not deleterious to the function of proteins as compared to both of its bisexual ancestors and that the two alleles which *P*. *formosa* received from its ancestors are likely to be equally well suited to serve the biological function of this gene. In most occasions, AA substitutions inferred to deviate from neutral expectations were at highly conserved positions (see S2 Table), which is compatible with an assumed biological impact of the respective substitution. Nonetheless, such computationally inferred impacts only comprise a first hint on a functional impact. Ultimately, sophisticated molecular modelling and/or biochemical/genetic experiments (e.g., mutagenesis) would be necessary to verify the impact of a substitution. Future experimental protein function studies could further investigate, how this is related to the gynogenetic reproductive mode of *P*. *formosa*. In this context, our study provides also SNP markers for allele specific expression research. Furthermore, the *P*. *mexicana* allele shows slightly less similarity in the NTD domain of Arα (cf. Fig 1), which is caused by the substitutions of positions 747, 919, 982 (cf. Fig 2). At those sites, *P*. *mexicana* was heterozygous. One of the nucleotides is the same as in the other species, the other one is different. If these are real SNPs within *P*. *mexicana*, they could constitute “derived” substitutions in the *P*. *mexicana* lineage. Alternatively, they may comprise ancestral lineage sorting in this bisexual species, i.e., an ancestral polymorphism that has been divergently sorted into two lineages (1) the sampled *P*. *mexicana* population and (2) the *P*. *formosa* lineage. Furthermore, we cannot fully exclude the possibility of an Taq amplification error here.

After the FSGD, the *arα* is secondarily lost in some teleost fish, e.g., zebra fish, Otophysi, and Salmoniformes, whereas the basal teleosts and percomorphs kept two copies of *ar*[64]. In our study, we observed a similar expression trend of *arα* and *arβ* only between ovary and brain, while expression in gill is different. This coincides with the gills not being involved in the brain-pituitary-gonadal axis, which is necessary for the reproduction and regulation of sex hormone [38], especially androgens. This phenomenon suggests the expression of *ar* genes to correlate with the amount and the location of androgen production.

Divergent evolution between *arα* and *arβ* is clearly illustrated by the large numbers of AA polymorphisms (cf. Fig 1). This could suggest that *arα* plays a more crucial role in the estradiol synthesis pathway, whereas the evolutionary pattern of *arβ* may indicate relaxed selection or even positive selection towards an alternate function, i.e., a neofunctionalization, as has been suggested for *arβ* in other teleost fish [64].

In the context of the evolution of genes interacting with the androgen pathway and involved in estrogen synthesis pathway in teleost fish, the aromatase has two conserved tissue-preferential isoforms, gonadal aromatase, preferentially expressed in ovary and encoded by the *cyp19a1* gene, and brain aromatase, preferentially expressed in the brain and encoded by the *cyp19a2*gene. Aromatase plays a pivotal role in sex determination and sex differentiation by controlling not only ovarian differentiation, but also testicular differentiation and brain sexual development in teleost fish by their up-regulation or down-regulation [65,66]. The strict ovary-specific *cyp19a1* and brain-specific *cyp19a2* expression reflects a common expression trend in teleost found in previous studies [67,68]. However, some studies demonstrate *cyp19a* expression not to be strictly limited to a specific tissue in some teleosts. A novel expression pattern of *cyp19a1* and *cyp19a2*, where both genes shifted the expression to testis tissue, was observed in Ectodine and Haplochromine cichlid fish [37]. Our expression data also show a deviation from a simple tissue-specific expression pattern. We found higher expression of *cyp19a1* in ovary as compared to brain (P< 0.05), and slightly higher expression of *cyp19a2* in brain as compared to ovary (except in *P*. *formosa*) (Fig 4). Taken together, *cyp19a1* and *cyp19a2* show deviation from strict tissue expression, i.e., *cyp19a1* is not only expressed in ovary and *cyp19a2* not only in brain, in agreement with other studies.

Estrogens have also been extensively studied as important hormones for ovarian differentiation in vertebrates [69–71], including teleost fish [72–75]. Interestingly, aromatase is also involved in estrogen synthesis by converting androgen to estrogen. The highly expressed *cyp19a2* is regulated by its promoter on EREs (estrogen response element) through the estrogen and aromatase feedback loop [33,67]. The expression pattern of *arα* and *cyp19a1* in ovary is similar among all three species. Hence, these two genes may be considered to be involved in the same pathway, which could be estrogen synthesis or other functions in the ovary. In a previous study, overexpressed *cyp19a2* of zebra fish embryo was observed in an estradiol treatment experiment and its induction was blocked by treatment with estradiol and ICI-182, 780 (ICI or Fulvestrant), an estrogen receptor (Er) antagonist. This suggests that Ers are involved in estradiol-dependent induction of the Cyp19a2 [76]. In our study, *erβ1* expression was not detected in gill and brain and extremely low in ovary across all three species. Consequently, we suggest that *erβ1* is not involved in the estradiol synthesis pathway at least in brain, where the aromatizable androgens could be aromatized to estrogen via Cyp19a. According to our expression data, *erα* may be more important in this context. In vivo and in vitro experimental approaches could confirm this interpretation.

The co-evolutionary relationship between Ar and aromatase was investigated in invertebrate and vertebrates including teleost fish [34,35]. Reitzel [34] demanded a clarification of gene orthology and appropriate background control genes for the analysis of co-evolutionary relationships among duplicated *ar* and pathway-related genes (for details see [34]). They also suggested alternative molecular or experimental approaches which could be combined with sequence divergence data to unravel the complicated evolutionary relationship between Ars and aromatases, both of which existing in duplicate in most teleost. We consider our combined sequence analysis and gene expression data to be potentially meaningful in this context, as the two paralogs *cyp19a1* and *cyp19a2* exhibit tissue-preferential expression Thus, we suggest that experimental approaches for illustrating the co-evolutionary relationship between *ar* and *cyp19a*s need to be specific with regard to both gene duplicates and tissues, as the different paralogs may be involved in different pathways within different tissue.

In conclusion, the heterozygous state of *ar*s gene and related pathway genes in *P*. *formosa* and its evolutionary relationship with its bisexual ancestors were confirmed in this study. The evolution of two paralogs of each gene, *arα* and *arβ*, *erα* and *erβ1*, *cyp19a1* and *cyp19a2*, were also investigated. Furthermore, we studied the distinct gene expression pattern found in various tissues. Taking into account the higher expression in ovary and brain than in gill, gene expression level of *ar*s may correlate with the amount and the location of androgen production. The expression pattern in *P*. *formosa* was conserved in those genes where expression did not differ between the two bisexual ancestors. In those cases, however, where gene expression differed between the two bisexual species, the *P*. *formosa* expression was always comparable to the higher expression of the two bisexual ancestors. This pattern does not indicate any significant deviation in gene expression between unisexual and bisexual mollies. Instead, it may reflect the hybrid nature of *P*. *formosa*: *P*. *formosa* has–for most genes–two different alleles, one of each of the two ancestors. As our expression analysis was so far not allele-specific, we could not confirm, whether similar expression levels between *P*. *formosa* and one of the two bisexual ancestors was caused by allele-specific expression differences among the two different alleles in the hybrid species *P*. *formosa*. Here, we suggest to utilize our allele-specific SNPs to establish a qPCR assay for the investigation of allele-specific expression in *P*. *formosa*.

Such studies should also involve specimens from different wild populations of the three species, in order to contrast among species effects with the within-species variability occurring in the wild.

## Supporting Information

S1 Fig Polymorphic nucleotide positions in the coding region of ERs and CYPAs. (PDF)

S1 File All gene pairwise comparisons. (PDF)

S2 File Relative expression analysis. (PDF)

S1 Table All Primers Information and NCBI GeneBank accession numbers. (PDF)

S2 Table Major AA frequencies in homologous GENBANK sequences for AA substitutions inferred to deviate from neutrality. (PDF)
