# Supplementary material for: Sequence Evolution and Expression of the Androgen Receptor and Other Pathway-Related Genes in a Unisexual Fish, the Amazon Molly, Poecilia formosa, and Its Bisexual Ancestors
Source: PLoS One. 2016 Jun 1;11(6):e0156209. doi: 10.1371/journal.pone.0156209 (PMC4889153; doi:10.1371/journal.pone.0156209)
Supplement: S1 Fig — (PDF) [file pone.0156209.s001.pdf]

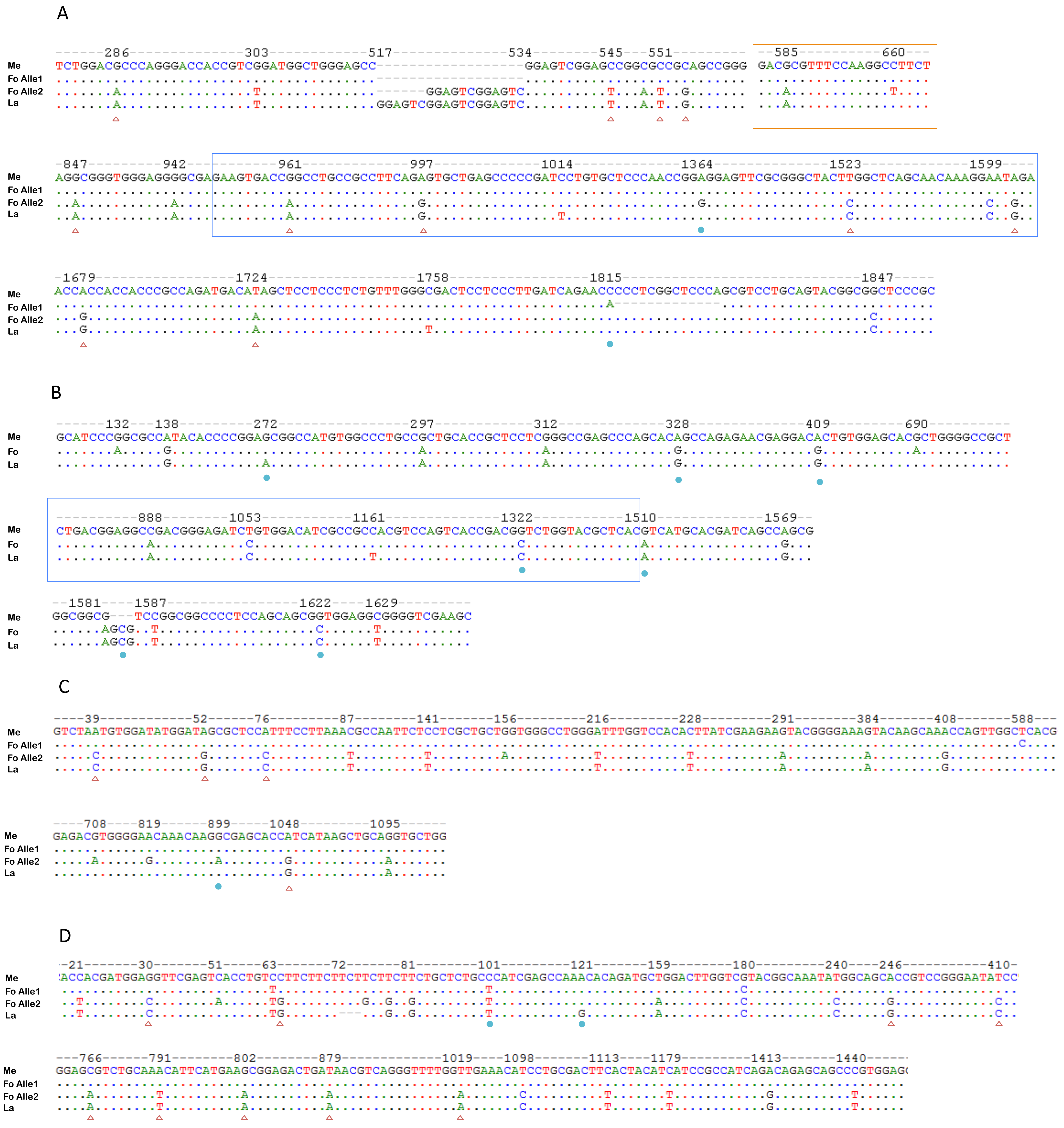


**S1 Fig. Polymorphic nucleotide positions in the coding region of ERs and CYPAs. A: ERa, B: ERb1, C: CYP19A1, D: CYP19A2**

Red triangles indicate non-synonymous substitutions among the ancestral alleles of P. mexicana and P. latipinna origin. The blue round point represents non-synonymous substitutions caused by mutation within a single lineage. Dots in the alignment indicate identity with P. mexicana. Colored boxes indicate different functional domains within the protein.
