# Supplementary material for: Sequence Evolution and Expression of the Androgen Receptor and Other Pathway-Related Genes in a Unisexual Fish, the Amazon Molly, Poecilia formosa, and Its Bisexual Ancestors
Source: PLoS One. 2016 Jun 1;11(6):e0156209. doi: 10.1371/journal.pone.0156209 (PMC4889153; doi:10.1371/journal.pone.0156209)
Supplement: S1 File — (PDF) [file pone.0156209.s002.pdf]

Supporting File 1 Indentity Matrix of Each Domain Amino Acids

1. NTD Percent Identity Matrix

1: Poecilia_mexicana_ARa_NTD	100.00	100.00	99.33	99.33	98.32	19.26	96.70	19.59	59.11	18.95	19.61	17.74	18.15	18.55	18.85	18.85	19.18	19.18
2: Poecilia_formosa_ARa_NTD_Alle1	100.00	100.00	99.33	99.33	98.32	19.26	96.70	19.59	59.11	18.95	19.61	17.74	18.15	18.55	18.85	18.85	19.18	19.18
3: Poecilia_formosa_ARa_NTD_Alle2	99.33	99.33	100.00	100.00	97.65	19.26	96.23	19.59	58.76	18.95	19.61	17.34	18.55	18.15	18.85	18.85	19.18	19.18
4: Poecilia_latipinna_ARa_NTD	99.33	99.33	100.00	100.00	97.65	19.26	96.23	19.59	58.76	18.95	19.61	17.34	18.55	18.15	18.85	18.85	19.18	19.18
5: Gambusia_affinis_ARa_NTD	98.32	98.32	97.65	97.65	100.00	30.92	98.49	31.11	69.44	29.86	31.06	29.23	29.23	29.23	18.29	18.29	18.62	18.62
6: Gambusia_affinis_ARb_NTD	19.26	19.26	19.26	19.26	30.92	100.00	32.36	99.21	31.28	64.36	40.99	35.75	36.12	35.60	98.73	98.73	98.47	98.47
7: Xiphophorus_hellerii_ARa_NTD	96.70	96.70	96.23	96.23	98.49	32.36	100.00	32.61	72.07	31.41	33.33	31.25	31.25	30.90	14.20	14.20	14.72	14.72
8: Xiphophorus_hellerii_ARb_NTD	19.59	19.59	19.59	19.59	31.11	99.21	32.61	100.00	31.20	64.75	41.74	35.67	36.04	35.53	98.98	98.98	98.73	98.73
9: Oreochromis_niloticus_ARa_NTD	59.11	59.11	58.76	58.76	69.44	31.28	72.07	31.20	100.00	29.67	30.33	32.05	30.96	31.78	18.60	18.60	18.52	18.52
10: Oreochromis_niloticus_ARb_NTD	18.95	18.95	18.95	18.95	29.86	64.36	31.41	64.75	29.67	100.00	40.86	35.85	36.66	35.50	58.61	58.61	58.61	58.61
11: Danio_rerio_AR_NTD	19.61	19.61	19.61	19.61	31.06	40.99	33.33	41.74	30.33	40.86	100.00	33.57	35.84	33.63	30.16	30.16	30.00	30.00
12: Sus_scrofa_AR_NTD	17.74	17.74	17.34	17.34	29.23	35.75	31.25	35.67	32.05	35.85	33.57	100.00	85.67	89.22	23.84	23.84	23.70	23.70
13: Rattus_norvegicus_AR_NTD	18.15	18.15	18.55	18.55	29.23	36.12	31.25	36.04	30.96	36.66	35.84	85.67	100.00	85.80	24.27	24.27	24.13	24.13
14: Human_AR_NTD	18.55	18.55	18.15	18.15	29.23	35.60	30.90	35.53	31.78	35.50	33.63	89.22	85.80	100.00	23.91	23.91	23.77	23.77
15: Poecilia_mexicana_ARb_NTD	18.85	18.85	18.85	18.85	18.29	98.73	14.20	98.98	18.60	58.61	30.16	23.84	24.27	23.91	100.00	100.00	99.75	99.75
16: Poecilia_formosa_ARb_NTD_Alle1	18.85	18.85	18.85	18.85	18.29	98.73	14.20	98.98	18.60	58.61	30.16	23.84	24.27	23.91	100.00	100.00	99.75	99.75
17: Poecilia_formosa_ARb_NTD_Alle2	19.18	19.18	19.18	19.18	18.62	98.47	14.72	98.73	18.52	58.61	30.00	23.70	24.13	23.77	99.75	99.75	100.00	100.00
18: Poecilia_latipinna_ARb_NTD	19.18	19.18	19.18	19.18	18.62	98.47	14.72	98.73	18.52	58.61	30.00	23.70	24.13	23.77	99.75	99.75	100.00	100.00

2. DBD Percent Identity Matrix

1: Poecilia_mexicana_ARa_DBD	100.00	97.56	97.56	97.56	97.56	69.51	97.50	69.51	95.12	69.51	69.51	67.07	67.07	67.07	69.51	69.51	69.51	69.51
2: Poecilia_formosa_ARa_DBD_Alle1	97.56	100.00	100.00	100.00	100.00	71.95	100.00	71.95	97.56	71.95	71.95	69.51	69.51	69.51	71.95	71.95	71.95	71.95
3: Poecilia_formosa_ARa_DBD_Alle2	97.56	100.00	100.00	100.00	100.00	71.95	100.00	71.95	97.56	71.95	71.95	69.51	69.51	69.51	71.95	71.95	71.95	71.95
4: Poecilia_latipinna_ARa_DBD	97.56	100.00	100.00	100.00	100.00	71.95	100.00	71.95	97.56	71.95	71.95	69.51	69.51	69.51	71.95	71.95	71.95	71.95
5: Gambusia_affinis_ARa_DBD	97.56	100.00	100.00	100.00	100.00	71.95	100.00	71.95	97.56	71.95	71.95	69.51	69.51	69.51	71.95	71.95	71.95	71.95
6: Gambusia_affinis_ARb_DBD	69.51	71.95	71.95	71.95	71.95	100.00	72.50	100.00	71.95	100.00	96.34	91.46	91.46	91.46	100.00	100.00	100.00	100.00
7: Xiphophorus_hellerii_ARa_DBD	97.50	100.00	100.00	100.00	100.00	72.50	100.00	72.50	97.50	72.50	72.50	70.00	70.00	70.00	72.50	72.50	72.50	72.50
8: Xiphophorus_hellerii_ARb_DBD	69.51	71.95	71.95	71.95	71.95	100.00	72.50	100.00	71.95	100.00	96.34	91.46	91.46	91.46	100.00	100.00	100.00	100.00
9: Oreochromis_niloticus_ARa_DBD	95.12	97.56	97.56	97.56	97.56	71.95	97.50	71.95	100.00	71.95	71.95	69.51	69.51	69.51	71.95	71.95	71.95	71.95
10: Oreochromis_niloticus_ARb_DBD	69.51	71.95	71.95	71.95	71.95	100.00	72.50	100.00	71.95	100.00	96.34	91.46	91.46	91.46	100.00	100.00	100.00	100.00
11: Danio_rerio_AR_DBD	69.51	71.95	71.95	71.95	71.95	96.34	72.50	96.34	71.95	96.34	100.00	90.24	90.24	90.24	96.34	96.34	96.34	96.34
12: Sus_scrofa_AR_DBD	67.07	69.51	69.51	69.51	69.51	91.46	70.00	91.46	69.51	91.46	90.24	100.00	100.00	100.00	91.46	91.46	91.46	91.46
13: Rattus_norvegicus_AR_DBD	67.07	69.51	69.51	69.51	69.51	91.46	70.00	91.46	69.51	91.46	90.24	100.00	100.00	100.00	91.46	91.46	91.46	91.46
14: Human_AR_DBD	67.07	69.51	69.51	69.51	69.51	91.46	70.00	91.46	69.51	91.46	90.24	100.00	100.00	100.00	91.46	91.46	91.46	91.46
15: Poecilia_mexicana_ARb_DBD	69.51	71.95	71.95	71.95	71.95	100.00	72.50	100.00	71.95	100.00	96.34	91.46	91.46	91.46	100.00	100.00	100.00	100.00
16: Poecilia_formosa_ARb_DBD_Alle1	69.51	71.95	71.95	71.95	71.95	100.00	72.50	100.00	71.95	100.00	96.34	91.46	91.46	91.46	100.00	100.00	100.00	100.00
17: Poecilia_formosa_ARb_DBD_Alle2	69.51	71.95	71.95	71.95	71.95	100.00	72.50	100.00	71.95	100.00	96.34	91.46	91.46	91.46	100.00	100.00	100.00	100.00
18: Poecilia_latipinna_ARb_DBD	69.51	71.95	71.95	71.95	71.95	100.00	72.50	100.00	71.95	100.00	96.34	91.46	91.46	91.46	100.00	100.00	100.00	100.00

3. LBD Percent Identity Matrix

1: Poecilia_mexicana_ARa_LBD	100.00	100.00	99.59	100.00	100.00	72.13	100.00	72.13	88.89	69.26	70.33	64.34	64.34	63.52	72.13	72.13	72.13	72.13
2: Poecilia_formosa_ARa_LBD_Alle1	100.00	100.00	99.59	100.00	100.00	72.13	100.00	72.13	88.89	69.26	70.33	64.34	64.34	63.52	72.13	72.13	72.13	72.13
3: Poecilia_formosa_ARa_LBD_Alle2	99.59	99.59	100.00	99.59	99.59	71.72	99.59	71.72	88.89	68.85	69.92	63.93	63.93	63.11	71.72	71.72	71.72	71.72
4: Poecilia_latipinna_ARa_LBD	100.00	100.00	99.59	100.00	100.00	72.13	100.00	72.13	88.89	69.26	70.33	64.34	64.34	63.52	72.13	72.13	72.13	72.13
5: Gambusia_affinis_ARa_LBD	100.00	100.00	99.59	100.00	100.00	72.13	100.00	72.13	88.89	69.26	70.33	64.34	64.34	63.52	72.13	72.13	72.13	72.13
6: Gambusia_affinis_ARb_LBD	72.13	72.13	71.72	72.13	72.13	100.00	72.13	100.00	68.10	91.43	86.07	72.43	72.43	71.60	100.00	100.00	100.00	100.00
7: Xiphophorus_hellerii_ARa_LBD	100.00	100.00	99.59	100.00	100.00	72.13	100.00	72.13	88.89	69.26	70.33	64.34	64.34	63.52	72.13	72.13	72.13	72.13
8: Xiphophorus_hellerii_ARb_LBD	72.13	72.13	71.72	72.13	72.13	100.00	72.13	100.00	68.10	91.43	86.07	72.43	72.43	71.60	100.00	100.00	100.00	100.00
9: Oreochromis_niloticus_ARa_LBD	88.89	88.89	88.89	88.89	88.89	68.10	88.89	68.10	100.00	65.52	63.25	56.90	56.90	56.03	68.10	68.10	68.10	68.10
10: Oreochromis_niloticus_ARb_LBD	69.26	69.26	68.85	69.26	69.26	91.43	69.26	91.43	65.52	100.00	84.02	69.55	69.55	68.72	91.43	91.43	91.43	91.43
11: Danio_rerio_AR_LBD	70.33	70.33	69.92	70.33	70.33	86.07	70.33	86.07	63.25	84.02	100.00	71.31	71.31	70.49	86.07	86.07	86.07	86.07
12: Sus_scrofa_AR_LBD	64.34	64.34	63.93	64.34	64.34	72.43	64.34	72.43	56.90	69.55	71.31	100.00	100.00	99.19	72.43	72.43	72.43	72.43
13: Rattus_norvegicus_AR_LBD	64.34	64.34	63.93	64.34	64.34	72.43	64.34	72.43	56.90	69.55	71.31	100.00	100.00	99.19	72.43	72.43	72.43	72.43
14: Human_AR_LBD	63.52	63.52	63.11	63.52	63.52	71.60	63.52	71.60	56.03	68.72	70.49	99.19	99.19	100.00	71.60	71.60	71.60	71.60
15: Poecilia_mexicana_ARb_LBD	72.13	72.13	71.72	72.13	72.13	100.00	72.13	100.00	68.10	91.43	86.07	72.43	72.43	71.60	100.00	100.00	100.00	100.00
16: Poecilia_formosa_ARb_LBD_Alle1	72.13	72.13	71.72	72.13	72.13	100.00	72.13	100.00	68.10	91.43	86.07	72.43	72.43	71.60	100.00	100.00	100.00	100.00
17: Poecilia_formosa_ARb_LBD_Alle2	72.13	72.13	71.72	72.13	72.13	100.00	72.13	100.00	68.10	91.43	86.07	72.43	72.43	71.60	100.00	100.00	100.00	100.00
18: Poecilia_latipinna_ARb_LBD	72.13	72.13	71.72	72.13	72.13	100.00	72.13	100.00	68.10	91.43	86.07	72.43	72.43	71.60	100.00	100.00	100.00	100.00
