# Supplementary material for: Sequence Evolution and Expression of the Androgen Receptor and Other Pathway-Related Genes in a Unisexual Fish, the Amazon Molly, Poecilia formosa, and Its Bisexual Ancestors
Source: PLoS One. 2016 Jun 1;11(6):e0156209. doi: 10.1371/journal.pone.0156209 (PMC4889153; doi:10.1371/journal.pone.0156209)
Supplement: S2 File — (PDF) [file pone.0156209.s003.pdf]

qRT-PCR experiment on *Poecilia formosa*, *Poecilia mexicana* and *Poecilia latipinna*

Genes tested:

ARα ($E_{AR\alpha}$=1.960)

ARβ ($E_{AR\beta}$=1.920)

CYP19A1 ($E_{CYP19A1}$=1.900)

CYP19A2 ($E_{CYP19A2}$=2.000)

ERα ($E_{ER\alpha}$ =1.963)

ERβ1 ($E_{ER\beta1}$=1.930)

Reference gene:

RPL7 (E_{RPL7}=1.931)

The formula for gene expression calculation:

$$\text{ratio} = \frac{(E_{\text{target}})^{\Delta CP_{\text{target}}(control-sample)}}{(E_{\text{ref}})^{\Delta CP_{\text{ref}}(control-sample)}}$$

Excluded samples when Ct mean >35.

Abbreviations used:

N=individuals for each biological group

RQ= relative quantification fold

SD=standard division

SE=standard error

CI=confidence interval

Gene Name:ARα

The mean value of each biological group

	Biological_Group	N	RQ	SD	SE	CI
1	Brain_Fo	4	0,50075	0,03872	0,01936	0,06162
2	Brain_La	7	0,72029	0,21138	0,07990	0,19550
3	Brain_Me	4	0,59185	0,15838	0,07919	0,25202
4	Gill_Fo	4	0,26355	0,13889	0,06944	0,22100
5	Gill_La	7	0,18230	0,04513	0,01706	0,04174
6	Gill_Me	4	0,16463	0,04835	0,02417	0,07693
7	Ovary_Fo	4	1,38038	0,44546	0,22273	0,70883
8	Ovary_La	6	2,07203	1,37660	0,56199	1,44465
9	Ovary_Me	3	1,05800	0,12302	0,07103	0,30560

Significant Wilcoxon Signed-Rank test results

	Group1	Group2	W	P_value	Null_value
[1,]	Gill_Me	Brain_Me	16	0,02857	0
[2,]	Ovary_Fo	Gill_Fo	0	0,02857	0
[3,]	Ovary_Fo	Brain_Fo	0	0,02857	0
[4,]	Ovary_La	Gill_La	0	0,00117	0
[5,]	Gill_La	Brain_La	49	0,00058	0

Gene Name: ARβ

The mean value of each biological group

	Biological_Group	N	RQ	SD	SE	CI
1	Brain_Fo	3	0,45193	0,13778	0,07955	0,34226
2	Brain_La	7	0,43781	0,12748	0,04818	0,11790
3	Brain_Me	4	0,41735	0,06173	0,03086	0,09822
4	Gill_Fo	4	0,09535	0,02975	0,01487	0,04734
5	Gill_La	7	0,11080	0,03791	0,01433	0,03506
6	Gill_Me	4	0,02635	0,00566	0,00283	0,00901
7	Ovary_Fo	4	1,54203	0,71314	0,35657	1,13477
8	Ovary_La	6	1,31193	0,61574	0,25137	0,64617
9	Ovary_Me	3	1,25643	0,47806	0,27601	1,18757

Significant Wilcoxon Signed-Rank test results

	Group1	Group2	W	P_value	Null_value
[1,]	Gill_Me	Brain_Me	16	0,02857	0
[2,]	Ovary_Fo	Gill_Fo	0	0,02857	0
[3,]	Ovary_La	Gill_La	0	0,00117	0

[4,]	Ovary_La	Brain_La	0	0,00117	0
[5,]	Gill_La	Brain_La	49	0,00058	0
[6,]	Gill_Me	Gill_La	28	0,00606	0
[7,]	Gill_Me	Gill_Fo	16	0,02857	0

Gene Name:ERα

The mean value of each biological group

	Biological_Group	N	RQ	SD	SE	CI
1	Brain_Fo	4	0,50508	0,31780	0,15890	0,50570
2	Brain_La	6	0,99163	0,58887	0,24040	0,61798
3	Brain_Me	4	0,91998	0,67697	0,33848	1,07721
4	Gill_Fo	3	0,12053	0,04620	0,02668	0,11478
5	Gill_La	7	0,02064	0,00904	0,00342	0,00836
6	Gill_Me	4	0,04728	0,02871	0,01436	0,04569
7	Ovary_Fo	4	0,73183	0,21327	0,10664	0,33937
8	Ovary_La	6	1,58368	1,14988	0,46944	1,20673
9	Ovary_Me	3	0,84610	0,13674	0,07895	0,33968

Significant Wilcoxon Signed-Rank test results

	Group1	Group2	W	P_value	Null_value
[1,]	Gill_Me	Brain_Me	16	0,02857	0
[2,]	Ovary_La	Gill_La	0	0,00117	0
[3,]	Gill_La	Brain_La	42	0,00117	0
[4,]	Gill_Me	Gill_La	2	0,02424	0
[5,]	Gill_La	Gill_Fo	21	0,01667	0

Gene Name:ERβ1

The mean value of each biological group

	Biological_Group	N	RQ	SD	SE	CI
1	Ovary_Fo	4	1,02073	0,38358	0,19179	0,61036
2	Ovary_La	6	1,92475	1,12879	0,46082	1,18459
3	Ovary_Me	3	1,17490	0,37409	0,21598	0,92928

Gene Name:CYP19A1

The mean value of each biological group

	Biological_Group	N	RQ	SD	SE	CI

1	Brain_Fo	4	0,02933	0,00330	0,00165	0,00525
2	Brain_La	6	0,02018	0,00559	0,00228	0,00586
3	Brain_Me	4	0,04643	0,01738	0,00869	0,02765
4	Ovary_Fo	4	1,85063	1,64768	0,82384	2,62182
5	Ovary_La	6	2,90587	2,74531	1,12077	2,88103
6	Ovary_Me	3	1,68183	1,23137	0,71093	3,05888

Significant Wilcoxon Signed-Rank test results

	Group1	Group2	W	P_value	Null_value
[1,]	Ovary_Fo	Brain_Fo	0	0,02857	0
[2,]	Ovary_La	Brain_La	0	0,00216	0
[3,]	Brain_Me	Brain_La	0	0,00952	0
[4,]	Brain_La	Brain_Fo	23	0,01905	0

Gene Name:CYP19A2

The mean value of each biological group

	Biological_Group	N	RQ	SD	SE	CI
1	Brain_Fo	4	2,32148	1,37331	0,68666	2,18525
2	Brain_La	6	6,00737	4,44236	1,81359	4,66197
3	Brain_Me	3	6,60433	5,19405	2,99878	12,90273
4	Gill_Fo	4	0,07433	0,02267	0,01133	0,03607
5	Gill_La	6	0,14088	0,08544	0,03488	0,08966
6	Gill_Me	4	0,13318	0,07903	0,03952	0,12576
7	Ovary_Fo	4	4,75045	1,12262	0,56131	1,78634
8	Ovary_La	6	3,43557	1,28117	0,52303	1,34450
9	Ovary_Me	3	1,58080	1,00623	0,58095	2,49963

Significant Wilcoxon Signed-Rank test results

	Group1	Group2	W	P_value	Null_value
[1,]	Ovary_Fo	Gill_Fo	0	0,02857	0
[2,]	Gill_Fo	Brain_Fo	16	0,02857	0
[3,]	Ovary_La	Gill_La	0	0,00216	0
[4,]	Gill_La	Brain_La	36	0,00216	0
[5,]	Ovary_Me	Ovary_La	17	0,04762	0

The ct value of all genes

PCR Eff		1.960	1.920	1.963	1.930	1.900	2.000	1.931
**Biological Group**	**Sample**	**ARa mean**	**ARb mean**	**ERa mean**	**ERb1 mean**	**CYPA1 mean**	**CYPA2 mean**	**RPL7 mean**
Ovary Me	Me6 ovary	26.893	26.071	27.486	30.506	26.507	26.604	20.543
Ovary Me	Me4 ovary	26.320	24.850	27.633	30.322	24.425	26.310	20.233
Ovary Me	Me3 ovary	29.410	31.938	31.421	NA	34.670	NA	21.190
Ovary Me	Me2 ovary	27.056	26.260	27.942	29.914	26.731	25.269	20.671
Ovary La	La4 ovary	NA	NA	NA	NA	NA	NA	25.937
Ovary La	La3 ovary	26.077	25.035	29.352	29.934	26.090	24.335	20.482
Ovary La	La2 ovary	26.410	25.941	26.782	29.593	25.882	25.848	20.053
Ovary La	La15 ovary	24.234	25.285	25.221	27.705	23.313	23.919	19.456
Ovary La	La13 ovary	23.244	23.320	24.135	27.036	21.700	23.124	19.034
Ovary La	La12 ovary	24.811	24.591	26.266	29.337	24.298	23.414	19.467
Ovary La	La1 ovary	28.059	26.515	27.494	30.628	26.492	25.221	20.752
Ovary Fo	Fo5 ovary	25.867	24.645	27.454	30.872	24.474	24.863	20.572
Ovary Fo	Fo3 ovary	27.141	26.233	28.719	30.195	27.671	24.797	20.923
Ovary Fo	Fo2 ovary	25.295	24.049	26.700	29.062	23.448	22.877	19.061
Ovary Fo	Fo1 ovary	27.237	26.793	28.773	31.726	28.918	24.700	21.288
Gill Me	Me6 gill	28.727	30.481	31.447	NA	NA	28.658	19.701
Gill Me	Me4 gill	29.680	32.370	31.649	NA	NA	29.254	21.146
Gill Me	Me3 gill	29.355	31.761	32.458	NA	NA	30.689	20.192
Gill Me	Me2 gill	29.746	31.063	32.190	NA	NA	29.384	20.129
Gill La	La4 gill	29.662	29.151	33.650	NA	NA	29.464	20.721
Gill La	La3 gill	28.820	28.609	33.925	NA	NA	30.384	19.721
Gill La	La2 gill	28.920	29.264	32.521	NA	NA	30.562	19.701
Gill La	La15 gill	28.351	27.913	31.418	NA	NA	27.804	19.539
Gill La	La13 gill	28.192	28.720	32.054	NA	NA	28.555	19.740
Gill La	La12 gill	29.023	29.188	33.561	NA	NA	28.668	20.510

Gill La	La1 gill	29.111	29.602	32.369	NA	NA	31.637	19.621
Gill Fo	Fo5 gill	29.606	30.016	31.192	NA	NA	30.299	20.557
Gill Fo	Fo3 gill	29.917	29.794	34.175	NA	NA	31.404	21.232
Gill Fo	Fo2 gill	29.633	31.419	32.415	NA	NA	32.280	22.193
Gill Fo	Fo1 gill	29.955	30.854	30.885	NA	NA	30.623	21.352
Brain Me	Me6 brain	27.861	28.079	28.937	NA	31.900	23.534	21.145
Brain Me	Me4 brain	27.987	27.605	27.534	NA	32.630	25.682	21.029
Brain Me	Me3 brain	28.119	27.734	27.013	NA	31.680	24.603	20.796
Brain Me	Me2 brain	28.765	28.204	30.746	NA	31.258	27.444	21.117
Brain La	La4 brain	29.259	29.457	32.027	NA	NA	29.095	21.488
Brain La	La3 brain	28.633	28.513	30.753	NA	33.786	26.855	21.947
Brain La	La2 brain	27.919	27.527	27.583	NA	33.828	24.900	20.734
Brain La	La15 brain	27.057	27.490	27.537	NA	32.999	23.493	20.738
Brain La	La13 brain	27.341	26.938	27.185	NA	32.346	23.887	20.697
Brain La	La12 brain	27.053	27.324	26.530	NA	32.378	22.928	20.461
Brain La	La1 brain	27.992	27.843	29.444	NA	32.903	26.952	20.951
Brain Fo	Fo5 brain	28.674	27.610	29.855	NA	32.639	25.707	21.137
Brain Fo	Fo3 brain	28.578	28.632	30.764	NA	32.507	27.043	21.218
Brain Fo	Fo2 brain	29.361	32.590	29.440	NA	33.813	27.671	22.075
Brain Fo	Fo1 brain	28.240	27.497	28.200	NA	32.405	24.979	20.966

The sample ct value higher than 35 was omitted
