# Supplementary material for: Sequence Evolution and Expression of the Androgen Receptor and Other Pathway-Related Genes in a Unisexual Fish, the Amazon Molly, Poecilia formosa, and Its Bisexual Ancestors
Source: PLoS One. 2016 Jun 1;11(6):e0156209. doi: 10.1371/journal.pone.0156209 (PMC4889153; doi:10.1371/journal.pone.0156209)
Supplement: S1 Table — (PDF) [file pone.0156209.s004.pdf]

Primer Information

Gene	GeneBank Accession Number	Primers Name	Primer sequence(5'-3')	GC content(%)	PCR product length(bp)
Androgen Receptor alpha (ARα)	AB174849.1	ARα-seq-F	CCAAGGACGAGAGCCGAAGATG	-	2170
		ARα-seq-R	CAGGGTCCGATTGAAAGG		
		ARα-qPCR-F	CCACTATCAAGACTGAACCT	54.5	110
		ARα-qPCR-R	CACCTGACACACTCTCCT		
Androgen Receptor beta (ARβ)	AB182329.1	ARβ1st-seq-F	CCAAGCGAACTCTTTCTGGG	-	1500
		ARβ1st-seq-R	TGCGTGCTCCAAGAGTCATCC		
		ARβ2nd-seq-F	AGTGGTATCCCGGCGGTATGCT	-	1360
		ARβ2nd-seq-R	GGACACGCCTGAAAGCAATC		
		ARβ-qPCR-F	GAAGAAGATTGGACAACAGAAGA	49.2	134
		ARβ-qPCR-R	ACTCAAGGATGTTCAGGAAGA		
Estrogen Receptor alpha (ERα)	XM_007547220.1	ERα-seq-F	CCGAGGATGATTCATGTATAAGAG	-	2003
		ERα-seq-R	CTTTCAAATCACTGCGTGTC		
	AB621910.1	ERα-qPCR-F	GAAGGAGACTGTGTTGAAG	55.8	129
		ERα-qPCR-R	AGAGTTGAGCAGGATGAT		
Estrogen Receptor beta 1 (ERβ1)	XM_007576835.1	ERβ1-seq-F	GGAGTAGCTGTCGTTTCCTG	-	1717
		ERβ1-seq-R	CAGTGAGATGTCGTCGGATC		
	AB295656.1	ERβ1-qPCR-F	GGAGCATCCAAGGTCATA	53	134
		ERβ1-qPCR-R	GATCCCACATTTGGTCAT		
Cytochrome P450, family 19, subfamily A, polypeptide 1(CYP19A1)	NW_0068000521	CYP19A1-seq-F	CGTTTTGTTCGCTCCGTCTT	-	1593
		CYP19A1-seq-R	GTGGACTTTGTTGGTGCGTC		
	DQ865279.1	CYP19A1-qPCR-F	CCATCAGCCTCTTCTTCA	54.4	101
		CYP19A1-qPCR-R	CATCTGTCTGTCGCCTAT		
Cytochrome P450, family 19, subfamily A, polypeptide 2(CYP19A2)	NW_006800423.1	CYP19A2-seq-F	CGCTTTGGACGAATAGAGGAGA	-	1805
		CYP19A2-seq-R	GGACTCGCGATTCTTTTCTGG		
	AY494837.1	CYP19A2-qPCR-F	GCTCTGGAGGACGATGACA	50	114
		CYP19A2-qPCR-R	GCTGAACTCTCTGGGTTTGG		
Ribosomal Protein L7 (RPL7)	XM_007554606.1	RPL7-F	TCAGAGGTATCAATGGTGTCCC	55.4	92
		RPL7-R	CAGCTTGACAAACACACCGT		

NCBI GeneBank accession numbers for phylogenetic tree construction

Species	Genebank accession number	Gene
Macaca_fascicularis	AM710396.1	AR
Homo_sapiens	M34233.1	AR
Danio_rerio	EU708622.1	AR
Sus_scrofa	NM_214314.2	AR
Bos_taurus	NM_001244127.1	AR
Chrysophrys_major	AB017158.1	AR
Rattus_norvegicus	NM_012502.1	AR
Gallus_gallus	NM_001040090.1	AR
Gambusia_holbrooki	DQ865278.1	ARa
Xiphophorus_hellerii	FJ372851.1	ARa
Oryzias_latipes	NM_001170833.1	ARa
Porichthys_notatus	JQ743204.1	ARa
Oreochromis_niloticus	NM_001279613.1	ARa
Gasterosteus_aculeatus	KF366305.1	ARa
Haplochromis_burtoni	NM_001286336.1	ARa
Gambusia_affinis	AB182328.1	ARa
Gasterosteus_aculeatus	KF366305.1	ARa
Xiphophorus_hellerii	FJ372852.1	ARb
Oreochromis_niloticus	NM_001279615.1	ARb
Gambusia_affinis	AB182329.1	ARb
Oryzias_latipes	AB252679.1	ARb
Rattus_norvegicus	NM_017085.2	CYP19A1
Oryzias_latipes	NM_001278879.1	CYP19A1
Oreochromis_niloticus	NM_001279586.1	CYP19A1
Bos_taurus	NM_174305.1	CYP19A1

Homo_sapiens	M22246.1	CYP19A1
Gallus_gallus	NM_001001761.2	CYP19A1
Danio_rerio	NM_131154.2	CYP19A1
Sus_scrofa	SSU52141.1	CYP19A1
Mus_musculus	EU252014.1	CYP19A1
Xiphophorus_maculatus	XM_005799744.1	CYP19A1
Gambusia_holbrooki	DQ865279.1	CYP19A1
Fundulus_heteroclitus	AY713118.1	CYP19A1
Chrysophrys_major	AB051290.1	CYP19A1
Gasterosteus_aculeatus	FJ773242.1	CYP19A1
Poecilia_reticulata	FJ236223.1	CYP19A1
Ophthalmotilapia_nasuta	KC684569.1	CYP19A1
Neolamprologus_pulcher	KC684565.1	CYP19A1
Limnotilapia_dardennii	KC684584.1	CYP19A1
Xiphophorus_maculatus	XM_005809885.1	CYP19A2
Poecilia_vivipara	KJ956688.1	CYP19A2
Fundulus_heteroclitus	AY428666.1	CYP19A2
Poecilia_reticulata	AY395692.1	CYP19A2
Oryzias_latipes	AY319970.1	CYP19A2
Oreochromis_niloticus	AF306786.1	CYP19A2
Danio_rerio	NM_131642.1	CYP19A2
Oryzias_sp.	D28954.1	ER
Chrysophrys_major	AB007453.1	ER
Homo_sapiens	NM_000125.3	ERa
Bos_taurus	NM_001001443.1	ERa
Rattus_norvegicus	NM_012689.1	ERa
Gallus_gallus	NM_205183.2	ERa
Mus_musculus	NM_007953.2	ERa
Danio_rerio	NM_152959.1	ERa
Sus_scrofa	NM_214220.1	ERa

Poecilia_reticulata	NM_001297487.1	ERa
Xiphophorus_maculatus	XM_005802592.1	ERa
Gambusia_affinis	AB295655.1	ERa
Fundulus_heteroclitus	AB097197.1	ERa
Haplochromis_burtoni	NM_001286335.1	ERa
Oreochromis_niloticus	NM_001279770.1	ERa
Porichthys_notatus	JF965426.1	ERa
Oreochromis_niloticus	NM_001279774.1	ERa
Homo_sapiens	NM_001437.2	ERb
Mus_musculus	NM_207707.1	ERb
Sus_scrofa	NM_001001533.1	ERb
Gallus_gallus	NM_204794.2	ERb
Fundulus_heteroclitus	AY570922.1	ERb
Oryzias_latipes	NM_001104702.1	ERb
Gambusia_affinis	AB295656.1	ERb1
Danio_rerio	CAC93848.1	ERb1
Gambusia_holbrooki	DQ865275.1	ERb1
Danio_rerio	NM_180966.2	ERb2
Gambusia_holbrooki	DQ865276.1	ERb2-like

Accesion Number Of Submitted Coding Sequences In NCBI : KP172520-KP172525, KT022990-KT023006
