# Supplementary material for: Sequence Evolution and Expression of the Androgen Receptor and Other Pathway-Related Genes in a Unisexual Fish, the Amazon Molly, Poecilia formosa, and Its Bisexual Ancestors
Source: PLoS One. 2016 Jun 1;11(6):e0156209. doi: 10.1371/journal.pone.0156209 (PMC4889153; doi:10.1371/journal.pone.0156209)
Supplement: S2 Table — (PDF) [file pone.0156209.s005.pdf]

Major AA frequencies in homologous GENBANK sequences for AA substitutions inferred to deviate from neutrality

Gene	Position	No of compared sequences	Major Amino Acid	AA1 Poecilia	AA2 Poecilia	AA1%	AA2%	Polyphen2 Prediction	PROVEAN Prediction	Code
ARa	307	149	C	C	R	100%	0%	Probably damaging	Deleterious	C307R
ARa	328	149	F	F	L	100%	0%	Probably damaging	Deleterious	F328L
ARa	454	142	Q	Q	L	97,9%	1,4%	BENIGN	Deleterious	Q454L
ERa	455	423	E	E	G	74,2%	0%	Probably damaging	Deleterious	E455G
ERa	508	411	S	S	L	41,4%	0,5%	BENIGN	Deleterious	S508L
ERa	534	406	M	M	I	90,4%	4,7%	BENIGN	Deleterious	M534I
ERb1	441	230	A	A	G	96,5%	1,3%	Probably damaging	Deleterious	A441G
ERb1	504	149	I	I	V	34,2%	14,8%	Probably damaging	Neutral	I504V
CYPA1	56	231	G	G	D	92,6%	6,5%	Probably damaging	Deleterious	G56D

Note that AA 1 depicts the AA which was generally more frequent at that position, irrespective of whether this AA was found in alleles of P. mexicana or P. latipinna origin.

Percentages are provided for the frequency of the respective AA to occur at this position throughout homologous sequences in GENBANK
